# Decentralized community-integrated research sites drive higher randomization rates: insights from a large-scale neurodegenerative disease trial

**DOI:** 10.3389/fmed.2025.1623776

**Published:** 2025-08-19

**Authors:** Seyed-Mahdi Khaligh-Razavi, Tyler E. Miller, Ralph Passarella, Ahmad Namvargolian

**Affiliations:** ^1^Care Access Research LLC, Boston, MA, United States; ^2^Case Western Reserve University School of Medicine, Cleveland, OH, United States; ^3^University Hospitals Cleveland Medical Center, Cleveland, OH, United States

**Keywords:** community-integrated research sites, decentralized clinical trials, neurodegenerative diseases, clinical trial recruitment, randomization rates, community engagement

## Abstract

**Introduction:**

Recruitment and retention remain critical challenges in clinical trials, particularly in neurodegenerative diseases, which require large participant populations, rigorous screening, and prolonged follow-up periods. Care Access is a global research site management organization that operates clinical trial sites employing various operational models. This study evaluates the operational performance of Care Access site models—including traditional sites, hub-and-spoke, and decentralized community-integrated research (DCIR) sites—within a Phase 3 neurodegenerative disease trial, focusing on their relative efficiency in recruitment, randomization, and retention. The inclusion of multiple site models within the same trial presents a rare opportunity for direct comparison under uniform study conditions, providing unique insights into their respective advantages and challenges. By analyzing key site performance metrics and the role of innovative operational strategies, this study aims to identify effective approaches to enhancing trial efficiency and overcoming recruitment challenges to inform the design and conduct of future trials.

**Methods:**

The trial involved 32 Care Access sites each employing one of these distinct operational models. Key performance metrics, such as participant screening rates, randomization rates, screen failure rates, and post-randomization discontinuation rates, were analyzed across (a) traditional, (b) hub-and-spoke, and (c) DCIR site models. We also compared the enrollment performance of Care Access to that of 196 non-Care Access sites using publicly available data.

**Results:**

DCIR Sites demonstrated the highest recruitment efficiency, screening 20.61 participants per site per month and randomizing 0.79 participants per site per month, compared to 11.78 and 0.50 for traditional sites, and 12.20 and 0.45 for hub-and-spoke sites, respectively. Despite being newly established, and operating in a decentralized model, DCIR sites achieved post-randomization discontinuation rates (28.17%) comparable to those of traditional site models (26.28%), highlighting their effectiveness in maintaining participant engagement. All site models encountered high screen failure rates (~95%), consistent with Phase 3 trials for neurodegenerative diseases. Notably, a community-engaged, research-only facility achieved the lowest discontinuation rate (17.65%) among all sites, highlighting the potential of strong local engagement to significantly enhance retention and participation. Furthermore, when comparing Care Access sites with non-Care Access sites in this trial, Care Access sites achieved an average randomization rate of 15.6 participants per site, outperforming the 8.7 participants per site recorded by non-Care Access sites. Data quality, monitoring practices, and overall data integrity were consistent across all site models, supporting the reliability of findings across both decentralized and traditional approaches. This comparison highlights the effectiveness of the innovative operational framework and decentralized community engagement approach in overcoming traditional recruitment challenges and enhancing trial outcomes.

**Discussion:**

DCIR sites exhibited superior participant screening and randomization efficiency while maintaining discontinuation rates comparable to traditional site models. This success was driven by a combination of innovative operational strategies, including decentralized community-based outreach mechanisms that expanded population access to research by bringing trials directly to populations that previously lacked access to clinical research. At the same time, this approach helped reach underrepresented groups, thereby improving both geographic coverage and trial generalizability while enhancing overall trial performance. Additionally, other innovations like the deployment of centralized remote research coordinators also played a role by streamlining remotely-conducted tasks, allowing site staff, in all site models, to focus on participant care and engagement. These findings highlight the effectiveness of a flexible, multi-model site strategy in addressing recruitment and retention challenges in large-scale Phase 3 neurodegenerative disease trials and suggest that this approach may extend to other therapeutic areas facing similar challenges.

## 1 Introduction

Recruitment efficiency in clinical trials has long been a significant bottleneck in drug development, delaying the delivery of critical therapies to participants and escalating overall trial costs. Over 80% of clinical trials experience delays due to recruitment challenges (1, 2), with nearly one-third of Phase 3 trials failing to meet enrollment targets. These challenges are particularly acute in most neurodegenerative disease trials, which necessitate large participant populations, rigorous screening, and sustained participant retention (3). Trials in these conditions face unique hurdles, including stringent eligibility requirements leading to high screen failure rates and lower retention rates due to lengthy follow-up periods (4).

Traditional clinical trial models, reliant on Brick-and-Mortar (B&M) sites, often exacerbate these inefficiencies. The fixed geographic reach of these sites limits access to diverse participant pools, while the necessity of in-person visits imposes logistical and time burdens on participants. These limitations highlight the need for innovative approaches to improve recruitment and retention while maintaining operational efficiency.

Recognizing these challenges, Care Access has developed clinical trial site models that combine decentralized trial components with localized community engagement. These models incorporate mobile infrastructure, community-based outreach, and centralized operational support to enhance participant access and alleviate site-level burdens. These approaches are designed to be responsive to trial demands and operational needs, allowing for adaptable resource allocation throughout the course of a study. By optimizing operations within an existing infrastructure, the model aims to address recruitment and retention challenges while maintaining trial continuity and efficiency. These strategies collectively represent a decentralized approach designed to function cohesively in addressing the complexities of clinical research.

Central to this model were standalone decentralized research sites specifically designed for this trial, combining flexibility and efficiency to overcome traditional recruitment and retention challenges. Additionally, these sites were supported by a team of centralized remote research coordinators, which offloaded certain site activities that could be conducted remotely. By streamlining these processes, this would allow clinical sites to focus on in-person participant activities, enhancing efficiency and increasing capacity during high recruitment periods.

In this study, we evaluate the performance of this new strategy through a neurodegenerative disease clinical trial, which required the enrollment of thousands of participants across diverse geographies. The trial posed significant recruitment and retention challenges, making it an ideal case study for assessing the efficacy of these site models and operational innovations. Notably, the inclusion of multiple site models—traditional (B&M), hub-and-spoke, and decentralized—within the same trial provides a rare opportunity for direct comparison under identical study conditions. This allows for a more rigorous evaluation of their relative strengths, limitations, and impact on trial outcomes. Our analysis highlights the ability of these models to maintain robust recruitment and retention rates while achieving operational efficiency.

Through this analysis, we aim to evaluate how a novel community-based decentralized model performed in terms of screening and randomization in comparison to traditional sites. This paper presents insights into how blending decentralized and community-focused strategies can address longstanding challenges in clinical trials, ultimately enabling faster delivery of therapies to patients in need.

## 2 Methods

### 2.1 Data

Data for the 32 Care Access sites were obtained from internal Care Access operational records. All detailed performance metrics—including screening rates, monthly randomization rates, screen failure rates, and discontinuation rates for Care Access sites—were derived entirely from internal operational records.

Enrollment performance at Care Access sites was also compared to that of 196 non-Care Access sites, using publicly available data from ClinicalTrials.gov, NCT05026866/TRAILBLAZER-ALZ 3. Specifically, we obtained the total number of non-Care Access sites and the total number of randomized participants to calculate average randomization rates. Detailed site-level metrics were not available for non-Care Access sites. As this comparison relies on aggregate data from ClinicalTrials.gov, it may be subject to delayed or inconsistent reporting.

The trial under study is a large-scale Phase 3 clinical trial for a neurodegenerative disease, enrolling a total of 2,196 participants. All performance metrics and comparisons are derived from this single multicenter Phase 3 trial, rather than from a pooled analysis of multiple studies. It included many sites across the United States, with a small number of international locations, and required screening and randomizing thousands of participants. Care Access sites were designed to operate alongside non-Care Access sites, providing an opportunity for direct performance comparison.

### 2.2 Site performance metrics

#### 2.2.1 Screening and randomization rates

Screening and randomization rates were key metrics used to evaluate site performance in this study. Screening rates were calculated by dividing the total number of participants screened at a site by the number of months the site was actively enrolling participants. This provides a standardized measure of screening efficiency over time, allowing for meaningful comparisons across sites with different enrollment durations. Similarly, randomization rates were calculated by dividing the total number of participants randomized at a site by the number of months it was open for enrollment. These rates reflect each site’s ability to enroll eligible participants during its enrollment-active period.

#### 2.2.2 Calculating average randomization rate for non-Care Access sites

Average randomization rates per site were calculated by dividing the total number of randomized participants by the number of sites for each group:

- Total randomized participants (sourced from ClinicalTrials.gov) = 2,196.
- Care Access sites: 498 randomized across 32 sites → 15.6 participants per site.
- Non-Care Access sites: (2196–498 = 1,698) randomized across 196 sites → 1,698/196 = 8.7 participants per site.

#### 2.2.3 Screen failure rate

The screen failure rate measures the proportion of screened participants who were deemed ineligible for randomization based on the trial’s inclusion and exclusion criteria. This rate was calculated by dividing the total number of screen failures by the total number of participants screened at a site. It serves as a key indicator of pre-screening effectiveness and the alignment of the screened population with the trial’s requirements.

#### 2.2.4 Post-randomization discontinuation rate

The post-randomization discontinuation rate captures the proportion of randomized participants who discontinued from the trial before its completion. This was calculated by dividing the total number of participants who discontinued after randomization by the total number of randomized participants. This metric reflects the trial’s ability to retain participants and maintain engagement throughout its duration, providing insights into retention strategies and their effectiveness.

It is important to note that some dropouts occur due to factors beyond the control of the site. These may include unexpected participant relocation, unforeseen health issues, or lack of continued interest in the study.

The study remains ongoing, with the enrollment period completed by mid-2024. Given that each participant in this trial is followed for approximately 3.5 years, additional discontinuations may still occur as participants continue through the remainder of their individual study timelines.

### 2.3 Centralized remote research coordinators

In this trial, centralized remote research coordinators were deployed to centralize certain site activities that could be conducted remotely, such as data entry, query resolution, and pre-screening. Offloading these administrative responsibilities from site staff, reduced logistical bottlenecks and allowed sites to focus more on participant care. This participant-centric approach enabled certain trial activities, such as pre-screening, consenting, and follow-up tasks, to be conducted remotely through telehealth, reducing the need for participant travel. Additionally, this approach expanded the capacity for pre-screening and participant randomization, facilitating smoother trial workflows and improving overall enrollment outcomes. The services provided by these centralized remote research coordinators were also referred to as the central clinical services (CCS).

#### 2.3.1 Key interventions by the centralized remote research coordinators

##### 2.3.1.1 Virtual pre-screening and consenting

Virtual pre-screening and consenting processes enable sites to efficiently identify potentially eligible participants remotely. This preparation helps make the required on-site visit faster, as much of the initial work is already completed remotely. This method streamlines administrative activities at the site level and accelerates participant identification, potentially enhancing overall enrollment speed and trial efficiency.

##### 2.3.1.2 Centralized data entry and query resolution

A centralized system for Electronic Source Documents and Electronic Data Capture (EDC) is implemented to handle data entry and resolve queries. This streamlined process can improve data quality by reducing site-level discrepancies and minimizing the time spent on back-and-forth clarifications with data management teams.

##### 2.3.1.3 Centralized study coordination

Centralized study coordinators manage scheduling of study visits, vendor coordination, and facilitation of assessments with central raters. This centralized coordination ensures consistency in study execution across sites and can reduce delays in participant visits and data collection, contributing to improved study timelines. Additionally, it frees up on-site staff to focus on patient-facing activities.

### 2.4 Care Access site models

Care Access is an organization that manages around 200 clinical research sites and operates about 150 mobile health clinics globally. It uses decentralized methods, including traveling clinical staff, to make it easier for people in different regions—including those that typically do not have access to clinical trials—to take part in research. A key part of its work involves ongoing community outreach to help increase access to studies and improve representation among participants.

#### 2.4.1 Traditional sites

**Figure 1:**
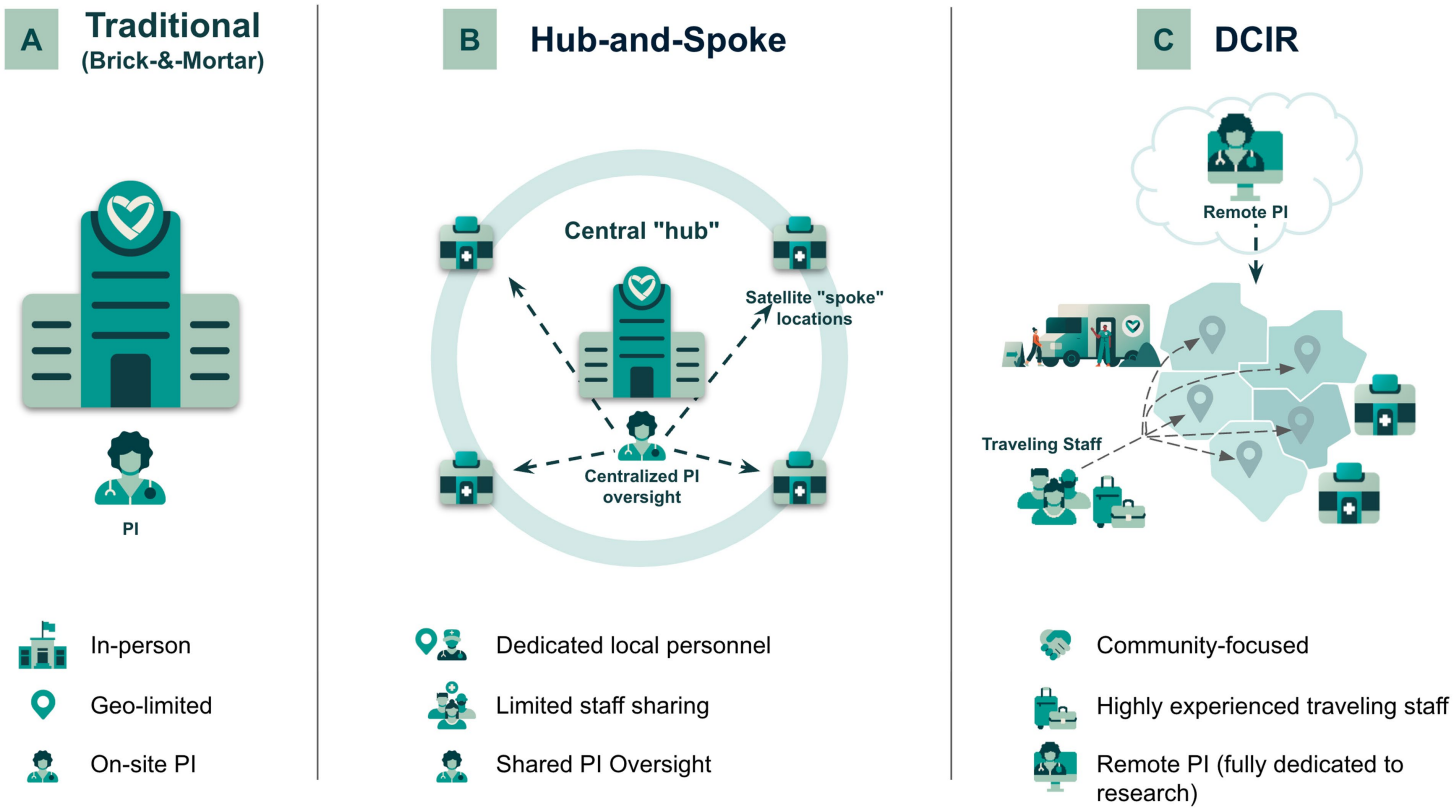
Comparison of Care Access site models. **(A)** Traditional (brick-and-mortar), **(B)** Hub-and-Spoke, and **(C)** Decentralized Community-Integrated Research (DCIR) site models are illustrated, highlighting differences in personnel structure, PI oversight, geographic reach, and the use of traveling staff to enhance community engagement and trial accessibility. Buildings with heart symbols represent Care Access physical (fixed) sites, while vehicles with heart symbols indicate mobile sites.

Traditional sites, also referred to as Brick-and-Mortar (B&M) sites, rely on fixed physical infrastructure (Figure 1A). In this model, all interactions, visits, and procedures occur in person, as is customary in conventional clinical trials. These sites are typically integrated into existing clinical practices, with dedicated research space either within or adjacent to clinical facilities, ensuring direct, face-to-face engagement between the Principal Investigator (PI), site staff, and participants. Trial activities, including participant assessments and visits, are conducted exclusively on-site, with a licensed Sub-Investigator (SubI), such as a Physician, Nurse Practitioner, or Physician’s Assistant, stepping in when the PI is unavailable. Due to their physical and operational structure, traditional sites have a geographically limited participant reach, requiring participants to reside near the site for regular in-person visits.

#### 2.4.2 Hub-and-spoke sites

Hub-and-spoke sites in this trial represented an evolution of traditional clinical trial models by incorporating selected elements of decentralization (Figure 1B). Each network consisted of a central “hub” site, typically adapted from a traditional clinical trial site, where an experienced PI was physically based. The PI remotely oversaw one or more satellite “spoke” locations, each staffed primarily by dedicated local personnel, with limited staff sharing across sites. This model blended remote activities such as pre-screening and questionnaires with essential in-person visits—including physical exams and infusions—to optimize operational efficiency, extend geographic reach, and enhance participant accessibility. The defining feature of this approach was the centralized PI oversight, enabling expertise from the central hub to effectively support satellite locations that otherwise might lack experienced local investigators.

#### 2.4.3 Decentralized community-integrated research (DCIR) sites

DCIR sites represent a modern, participant-centric clinical trial site model designed to enhance accessibility and operational flexibility (Figure 1C). These sites are typically stand-alone research sites strategically positioned to integrate into the community and leverage partnerships with local healthcare providers who traditionally have not been involved in research. These independent sites actively engage local participants, relying on mobile, community-focused infrastructure rather than serving as satellite extensions of a central hub.

Unlike traditional models, DCIR sites utilize highly experienced traveling clinical research staff who commute directly from their homes to the research locations, facilitating direct participant engagement and care, thus enabling the conduct of clinical research in a broader geographic reach. The goal is to integrate deeply into previously unreached communities to increase overall participant engagement. Traveling staff are specifically trained to rapidly adapt to diverse and often remote research environments, providing flexible and on-demand clinical expertise.

In short, key characteristics of DCIR sites include the establishment of local research hubs within underserved communities, the deployment of traveling clinical research teams to conduct on-site assessments and procedures, and remote oversight by dedicated PIs who manage multiple decentralized locations. These PIs are supported by traveling Sub-Investigators who conduct key investigator-level assessments on-site. DCIR sites feature significant operational flexibility, employing adaptable staffing and infrastructure that enable rapid scaling and efficient resource allocation to meet evolving trial needs.

#### 2.4.4 Community-integrated excellence: Care Access’s top performing research site (referred to as the innovation center)

**Figure 2:**
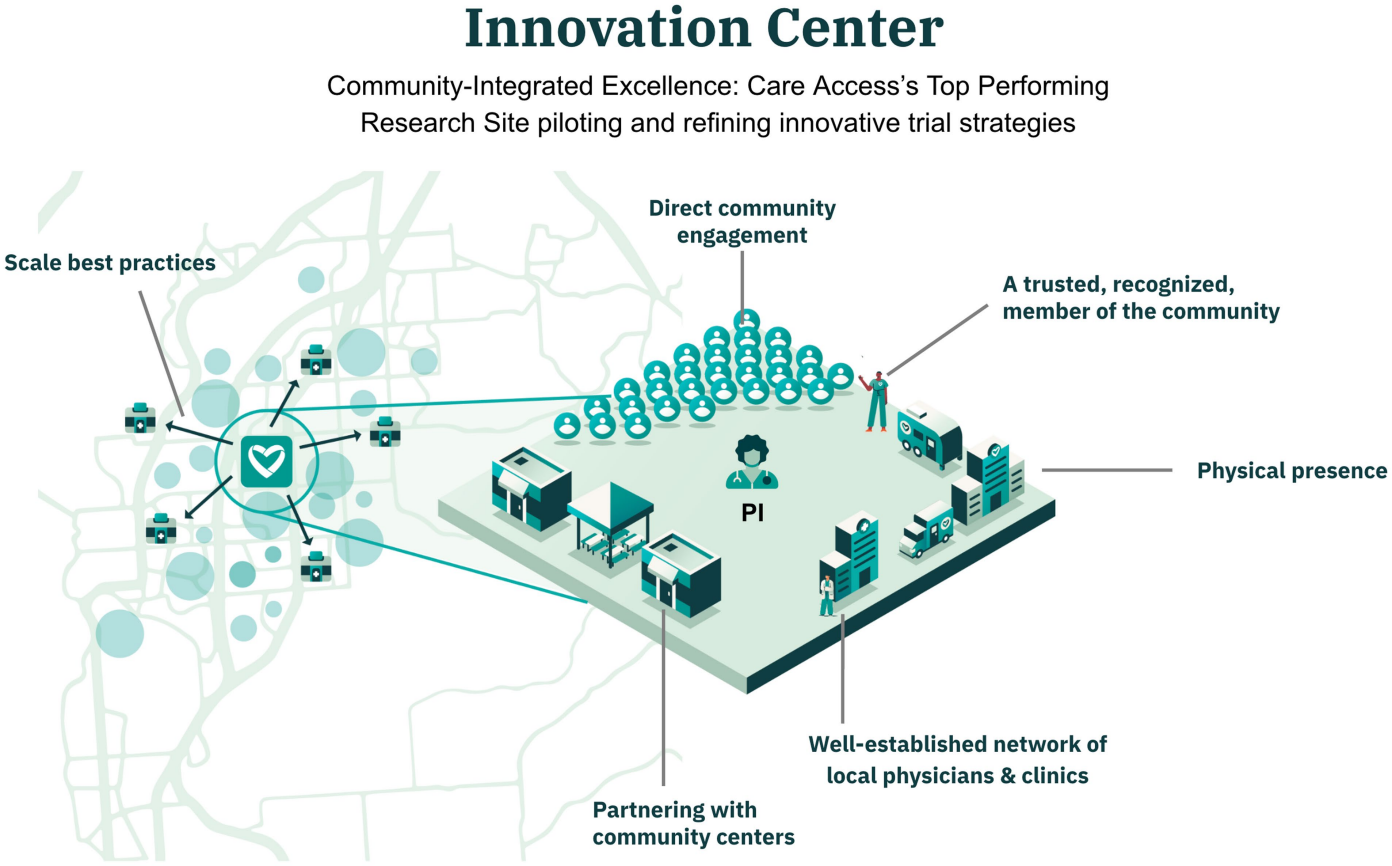
Innovation center as a community-integrated research hub. The Innovation Center is deeply embedded in the local healthcare landscape, partnering with community centers and clinics to pilot and refine innovative trial strategies. Its strong physician network, physical and digital presence, and trusted community role enable high participant engagement and the scaling of best practices across the Care Access network.

The top performing Care Access site, here referred to as the Innovation Center (Figure 2), was analyzed separately due to its unique community-integrated model. It was supported by a well-established network of local physicians built over a decade, which contributed significantly to its success in participant recruitment and retention. This site was a model for the community integrated research sites (i.e., DCIR), with the distinction that DCIR sites were newly established and mainly operated in a decentralized mode with traveling staff.

##### 2.4.4.1 Innovation hub and role model function

The Innovation Center functioned as an innovation hub, piloting and refining innovative trial strategies. In addition to serving as an operational site, it served as a role model and R&D center for other Care Access sites, acting as a frontier for testing and refining innovative strategies. Successful approaches piloted at this site were later adapted and scaled across the broader Care Access network, enhancing the overall efficiency and effectiveness of decentralized trial operations.

##### 2.4.4.2 Community engagement

The Innovation Center was deeply embedded within the local healthcare community, leveraging established relationships to engage and retain participants effectively. This strong community presence was instrumental in achieving high retention rates and lower screen failure rates.

### 2.5 Quality assurance and regulatory compliance

Care Access sites, including the newly established DCRI models have undergone quality assurance audits and regulatory inspections, including sponsor audits and FDA inspections. These evaluations have confirmed consistent compliance with Good Clinical Practice (GCP) guidelines and all applicable regulatory standards.

### 2.6 Effect size calculation: Cliff’s Delta

Cliff’s Delta is a non-parametric effect size measure used to assess the degree of overlap between two independent distributions. It provides insight into the relative positioning of values in two groups, offering a comparison without making assumptions about the data’s distribution. Cliff’s Delta is particularly useful in situations where the data may not follow a normal distribution or when group sizes are unequal.

The metric, Δ\Delta, is calculated by comparing each possible pair of observations across two groups:

- Positive pairs: Instances where a value from Group A is greater than a value from Group B.
- Negative pairs: Instances where a value from Group A is less than a value from Group B.
- Tied pairs: Instances where the values are equal.

Cliff’s Delta is computed using the formula:


$$\Delta =\frac{\text{Number of positive pairs}-\text{Number of negative pairs}}{\text{Total number of pairs}}$$

where the total number of pairs is the product of the sizes of the two groups (excluding any missing values).

The value of Cliff’s Δ ranges from −1 to 1:

- Δ = 1 → All values in Group A are greater than all values in Group B.
- Δ = −1 → All values in Group A are smaller than all values in Group B.
- Δ = 0 → Complete overlap between the two distributions, meaning no meaningful difference.

#### 2.6.1 Interpretation thresholds

To interpret the effect size, we apply the following thresholds following Romano et al. (5).

- Negligible: |Δ| < 0.147.
- Small: 0.147 ≤ |Δ| < 0.33.
- Medium: 0.33 ≤ |Δ| < 0.474.
- Large: |Δ| ≥ 0.474.

These thresholds help categorize the degree of difference between groups, providing context for whether the observed effect size is meaningful. In our study, Cliff’s Delta was calculated to compare screening and randomization rates across site models, enabling a clear assessment of whether one group consistently outperformed the other in terms of participant enrollment metrics.

## 3 Results

In this study, three distinct clinical trial site models were utilized to evaluate operational efficiency, participant recruitment, and retention strategies: Traditional (also referred to as Brick-and-Mortar or B&M), Hub-and-Spoke, and Decentralized Community-Integrated Research (DCIR) sites. Traditional sites conducted all interactions and procedures at fixed locations, limiting participation to individuals within geographic proximity. The Hub-and-Spoke model utilized a main site with a physically-present PI, managing satellite locations remotely with local staff support. DCIR sites represented a modern, participant-centric approach, integrating into local communities through dedicated traveling clinical staff and remote oversight by specialized principal investigators.

A notable example of the DCIR approach was demonstrated at the Innovation Center, which was a Care Access research site functioning as an innovation hub for piloting and refining participant recruitment strategies. Leveraging a community network established over a decade, the Innovation Center served as an R&D center to evaluate methods for participant recruitment, retention, and decentralized clinical trial operations. In this study, we assessed whether successful practices developed at the Innovation Center could be replicated and scaled across a broader network of DCIR sites, and explored the mechanisms facilitating this wider implementation.

### 3.1 Performance comparison across traditional research sites, hub-and-spoke, and decentralized community-integrated research (DCIR) sites within Care Access

The performance of Care Access site categories—traditional B&M, hub-&-spoke, and DCIR Sites—was evaluated across key metrics to understand the operational efficiency and recruitment outcomes of different site models (Table 1).

**Table 1:** Screening, retention, and randomization metrics by Care Access site model.

Site model	Discontinued before randomization	Screen failure rate (%)	Discontinued after randomization (count)	Discontinuation rate after randomization (%)	Number of sites	Total screened	Total randomized
DCIR	5,318	95.56	60	28.17	12	5,565	213
Traditional	2,672	95.36	31	26.28	11	2,802	118
Hub-and-Spoke	2058	95.15	23	28.05	8	2,163	82

This table summarizes key performance metrics for different Care Access site models in the trial. Metrics include screen failure rates, discontinuation rate after randomization, total participants screened, and total participants randomized. It also includes the number of sites and participants discontinued before and after randomization, providing insight into site-specific efficiencies in participant retention and randomization.

#### 3.1.1 Screening and randomization analysis

Traditional, hub-&-spoke and DCIR sites showed notable variations in screening and randomization efficiency. Across all Care Access site models, a total of 10,530 participants were screened, resulting in 413 randomized participants (Table 1).

DCIR sites accounted for the highest proportion of participants screened (53%), screening 5,565 participants across 12 sites. These sites also demonstrated the hallmark advantage of decentralized models with broader geographic reach. Of the participants screened at DCIR sites, 213 were randomized (52% of the total randomized participants), while 5,318 participants discontinued before randomization.

Traditional sites contributed to 27% of the total participants screened, with 2,802 participants screened across 11 sites. From this group, 118 participants were randomized (29% of the total randomized), while 2,672 participants discontinued before randomization.

Hub and spoke sites screened 2,163 participants, representing 20% of the total, and randomized 82 participants (20% of the total randomized) across the model. A total of 2,058 participants at hub-and-spoke sites discontinued before randomization.

This analysis highlights that DCIR sites lead in both screening and randomization volume, reflecting their effectiveness in decentralized trial operations.

#### 3.1.2 Screen failure rate across site models

Screen failure rates and post-randomization discontinuation rates are critical metrics of trial performance, reflecting the ability to accurately identify eligible participants and maintain their engagement throughout the trial. Screening failure rates were consistently high (~95%) across all site models, including traditional, hub-and-spoke, and DCIR sites, highlighting the challenges of early participant identification in clinical trials, particularly in neurodegenerative disorders.

Despite these challenges, the DCIR model, even with its decentralized structure and newly established sites, demonstrated screen failure rates comparable to those of traditional and hub and spoke sites. This underscores the ability of decentralized models to maintain pre-screening effectiveness while expanding geographic reach and flexibility. The Kruskal-Wallis test, a non-parametric comparison of screen failure rates across the three models, yielded a test statistic of 0.43 and a *p*-value of 0.81, indicating no statistically significant differences between the site models.

#### 3.1.3 Post-randomization discontinuation rates

Post-randomization discontinuation, or participant dropout, is a common challenge in longitudinal randomized controlled trials (RCTs). Dropout rates are often higher in specialized therapeutic areas such as neurodegenerative diseases, ranging from around 23% to higher dropouts in some studies (6–8). Contributing factors typically include adverse events (e.g., infusion-related reactions or amyloid-related imaging abnormalities), as well as participant-driven withdrawal resulting from disease progression and its associated burdens.

Here the post-randomization discontinuation rates showed minimal variation across the site models. Traditional sites reported a discontinuation rate of 26.28%, while DCIR and hub-and-spoke models reported rates of 28.17 and 28.05%, respectively. These findings suggest that decentralized models like DCIR, despite their remote and flexible nature, can achieve comparable retention outcomes to traditional B&M models. The Kruskal–Wallis test for discontinuation rates (statistic = 1.16, *p* = 0.56) confirmed no statistically significant differences among the site models. A review of 71 randomized controlled trials in four top medical journals showed dropout rates of 20% or more in 18% of the trials.

### 3.2 Screening and randomization rate by site model

**Figure 3:**
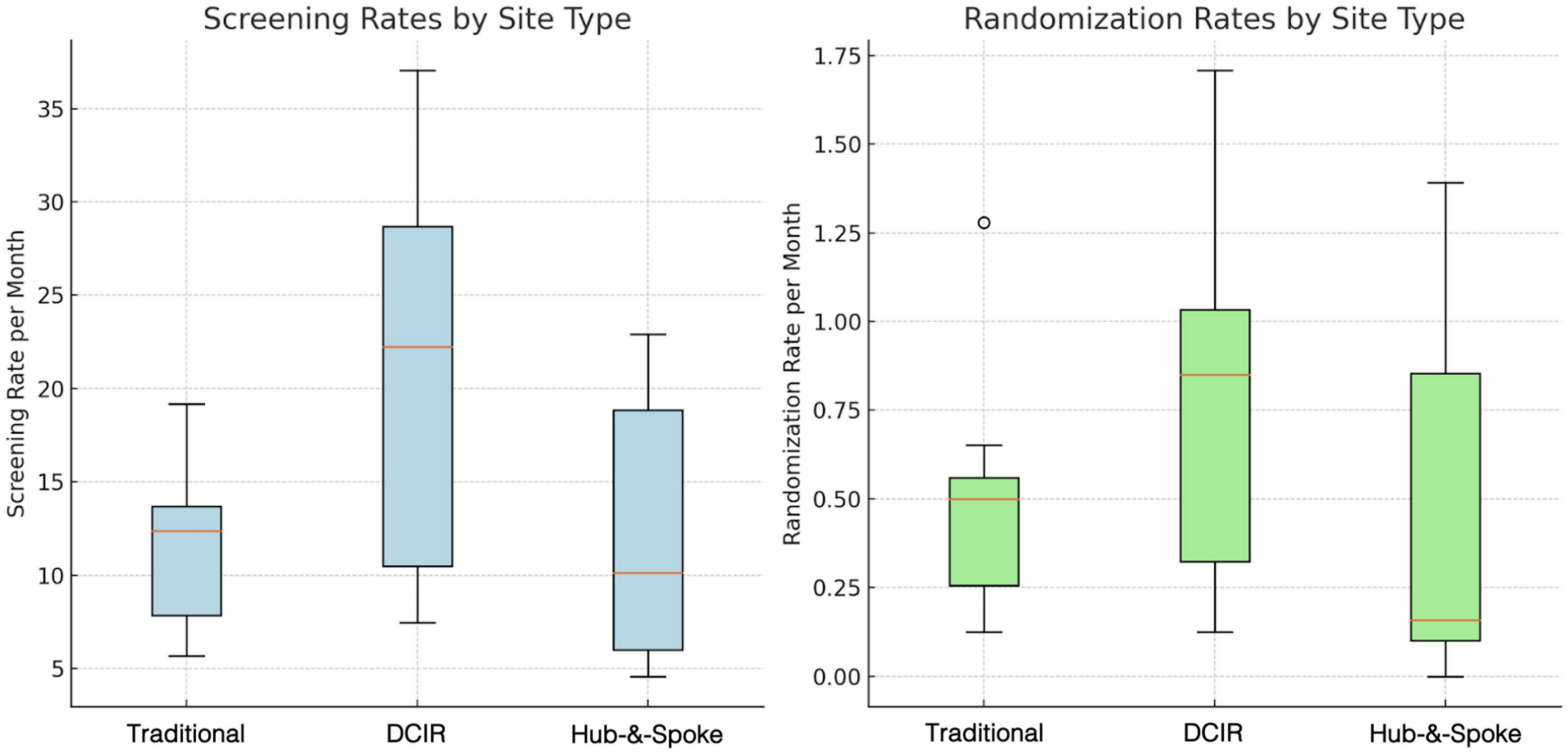
Participant screening and randomization rates by site model. Box plots comparing the screening rates (left, light blue) and randomization rates (right, light green) across three site models: traditional, hub and spoke and DCIR. Screening rates are calculated by dividing the total number of participants screened by the number of months the site has been active. Similarly, randomization rates are calculated by dividing the total number of participants randomized by the number of active months. The central line in each box represents the median, while the box edges denote the interquartile range (IQR). Whiskers extend to values within 1.5 times the IQR, with outliers shown beyond the whiskers.

The screening and randomization rate of each site model was analyzed by categorizing them into different groups based on their operational models: traditional, hub-and-spoke, and DCIR. Site screening and randomization rate was measured by the average number of participants screened per month and randomized per month for each site model (Table 2; Figure 3).

**Table 2:** Participant screening and randomization rates by site model.

Site model	Participants screened/Month	Participants randomized/Month	Number of sites	Number of PIs
DCIR	20.61	0.79	12	6
Traditional	11.78	0.50	11	10
Hub-and-Spoke	12.20	0.45	8	2

#### 3.2.1 Screening rates and effect sizes

DCIR sites achieved the highest average screening rate, screening 20.61 participants per month (Table 2; Figure 3), highlighting the potential of decentralized models to reach more participants through broader geographic access and remote screening capabilities. In comparison, traditional and hub-and-spoke sites screened 11.78 and 12.20 participants per month, respectively, suggesting that these models, while operationally consistent, may have a more localized participant reach.

**Figure 4:**
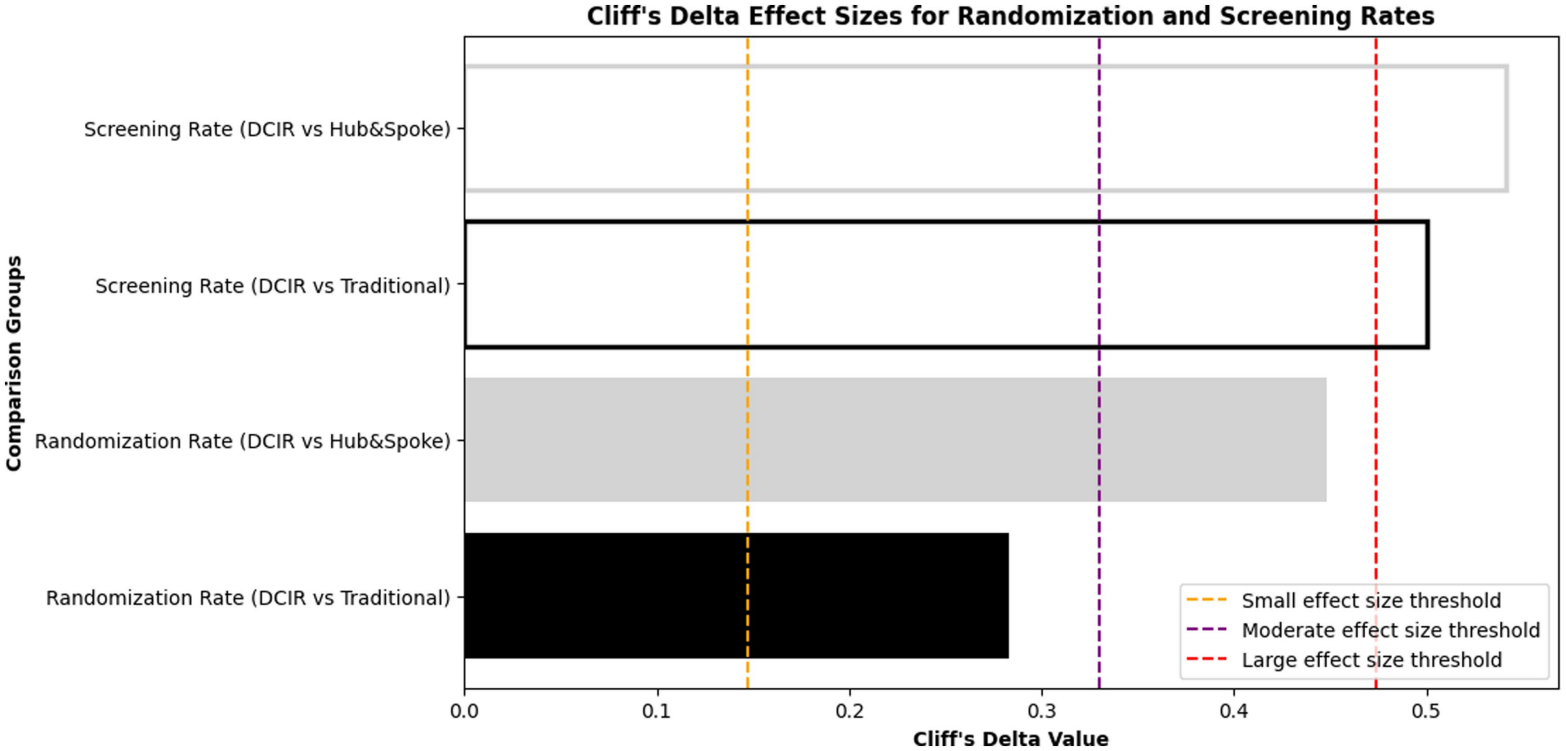
Cliff’s Delta effect sizes for randomization and screening rates between Care Access site models. Cliff’s Delta values are presented for comparisons of screening and randomization rates between DCIR sites and two other site models: traditional and hub-and-spoke. Screening Rate comparisons (DCIR vs. traditional and DCIR vs. hub-and-spoke) are shown as hollow bars, while Randomization Rate comparisons (DCIR vs. traditional and DCIR vs. hub-and-spoke) are displayed as filled bars. Vertical dashed lines represent effect size thresholds for interpretation: Negligible effect size: |Δ| < 0.147; Small effect: |Δ| = 0.147 to 0.32; Moderate effect: |Δ| = 0.33 to 0.473; Large effect: |Δ| ≥ 0.474.

Statistical analysis confirmed that DCIR sites had significantly higher screening rates than traditional and hub-and-spoke sites combined (*n* = 100,000, one-sided permutation test, *p* = 0.004). To assess the magnitude of these differences, we used Cliff’s Delta, a non-parametric effect size measure that quantifies the degree of overlap between two distributions. The analysis (Figure 4) demonstrated large effect sizes, reinforcing the practical significance of DCIR’s superior screening performance compared to both models.

#### 3.2.2 Randomization rates and effect sizes

DCIR sites exhibited the highest randomization rate, averaging 0.79 participants per month, underscoring the decentralized model’s ability to enhance participant engagement and support higher randomization rates (Table 2). In contrast, traditional and hub-and-spoke sites demonstrated lower average randomization rates of 0.50 and 0.45 participants per month, respectively, indicating a consistent yet comparatively lower performance.

Statistical analysis confirmed that DCIR sites had significantly higher randomization rates than traditional and hub-and-spoke sites combined (*n* = 100,000, one-sided permutation test, *p* = 0.044). Cliff’s Delta analysis (Figure 4) further explored the magnitude of these differences. Randomization rate comparisons, shown as filled bars, indicated small to moderate effect sizes, with DCIR sites performing better than the other models. These effect sizes suggest operational advantages for DCIR models in terms of randomization performance.

### 3.3 Site capacity enhanced by centralized remote research coordinators

The deployment of centralized remote research coordinators across all site models significantly enhanced the operational efficiency of sites in this trial. This centralized team expanded site capacity for pre-screening and participant randomization while also reducing administrative and logistical bottlenecks that typically impede site productivity.

During the screening and enrollment phases, Care Access utilized 149 clinical staff members (equivalent to 2,831 FTE-months), including 88 centralized remote study coordinators, 36 research assistants, 13 managers, 2 leads, and 1 trainer. This approach supported a participant-centric model, minimizing participant travel through the completion of tasks via telemedicine and thereby reducing participant burden.

Following the enrollment period, the centralized team demonstrated adaptability by adjusting staffing levels according to evolving trial requirements. The team was optimized to 97 staff members, delivering an additional 679 FTE-months of support for post-enrollment activities. Staff no longer needed for this trial were efficiently reallocated to other ongoing trials. This strategy ensured optimal resource use while maintaining high site functionality and seamless management of post-randomization tasks, highlighting the flexibility and scalability of the centralized support model.

The combined support of 3,510 FTE-months across approximately 200 sites (Care Access and non-Care Access sites) translates into an average of 17.6 months of full-time support per site. This underscores the increased capacity and sustained operational effectiveness provided by the centralized remote research coordinators throughout the duration of the study.

### 3.4 Case study of a high-performing community-integrated research site (i.e., innovation center)

#### 3.4.1 Overview of performance

A notable example of the DCIR approach was demonstrated at Care Access’s Innovation Center, which functioned as an innovation hub for piloting and refining participant recruitment strategies. Leveraging a community network established over a decade, The Innovation Center served as a research and development (R&D) center to implement new methods for running clinical research. In this study, we assessed whether successful practices developed at the Innovation Center could be replicated and scaled across a broader network of DCIR sites and explored the mechanisms facilitating this wider implementation.

The Innovation Center was analyzed separately due to its distinct community-integrated model, characterized by a well-established network of local physicians and strong community ties. These attributes set it apart from other sites and contributed to its success in participant recruitment, retention, and trial performance.

Unlike traditional sites, the Innovation Center did not function within a clinical practice setting but instead operated exclusively as a research site, dedicated to trial-related activities. It relied on community-based recruitment, and also benefited from EMRs from partner clinics to identify and engage participants. This model, which relied on robust local networks and collaborations with community physicians, demonstrated the potential for infrastructure that DCIR sites aim to develop over time.

The deep integration of the Innovation Center into the community, combined with the flexibility and recruitment strategies associated with decentralized models, contributed to its exceptional success in participant engagement and overall trial performance.

#### 3.4.2 Total participants screened

The Innovation Center screened 886 participants, demonstrating notably high efficiency (Table 3). This efficiency was primarily driven by its extensive experience, strong physician network, and deep community engagement. These factors also contributed to a lower screen failure rate compared to other Care Access sites.

**Table 3:** Key site performance metrics for the innovation center.

Site name	Participants screened/Month	Participants randomized/Month	Number of sites and PI	Screen failure rate (%)	Discontinuation rate after randomization (%)	Total screened	Discontinued before randomization	Discontinued after randomization	Total randomized
Innovation center	32.77	3.14	1	90.29	17.65	886	800	15	85

The decentralized community-integrated research site is a high-performing Care Access site.

#### 3.4.3 Screen failure and discontinuation rates

The Innovation Center (Table 3) showed the lowest screen failure rate at 90.29%, compared to the average of approximately 95% across other Care Access sites. This site also demonstrated a lower post-randomization discontinuation rate at 17.65%. This superior performance is attributed to the strong local ties and personalized care approach that were crucial in maintaining participant engagement throughout the trial.

### 3.5 Comparison of Care Access sites with non-Care Access sites

Using publicly available information about this trial from clinicaltrials.gov, which included basic trial metrics such as the total number of participants randomized and the total number of sites, we analyzed the average randomization rates of Care Access sites compared to non-Care Access sites. The findings reveal that Care Access sites outperformed their counterparts. Across Care Access sites, the average randomization rate was 15.6 participants per site—nearly double the 8.7 participants per site achieved by other sites.

These calculations were derived from the total trial randomization count of 2,196 participants, of which 498 were randomized at Care Access sites, divided by the number of sites (see methods for more details).

## 4 Discussion

This study examined the performance of different site models within Care Access (Traditional, hub-and-spoke, and DCIR sites), revealing distinct differences in recruitment efficiency, randomization rates, and operational agility based on the model used. These findings provide key insights into the relative strengths and challenges of each site model in the context of a Phase 3 neurodegenerative disease trial. The observed trends in recruitment, retention, and operational efficiency also have broader implications for other large-scale clinical trials with similar characteristics, such as those requiring extensive outreach, long follow-up periods, or complex eligibility criteria.

### 4.1 Recruitment and screening efficiency

DCIR sites exhibited the highest recruitment reach and screening efficiency, largely due to their ability to engage a broader geographic pool of participants (Table 2). This increased screening efficiency also translated into higher randomization rates in DCIR sites compared to other models, underscoring the operational advantages of decentralized approaches.

This also reflects the operational strength of DCIR models, which rely less on fixed physical infrastructure and instead emphasize geographic expansion by bringing research to communities previously lacking access to clinical research. This approach enhances participant engagement and broadens overall trial outreach.

### 4.2 Randomization and discontinuation rates

All three models—traditional, hub-and-spoke, and DCIR—faced similar challenges in progressing participants from screening to randomization, with high screen failure rates across both Care Access and non-Care Access sites. This was largely due to the stringent eligibility criteria inherent to most neurodegenerative disease trials and the complexity of pre-screening for a diverse population. Notably, there was no statistically significant difference in screen failure rates among the models, indicating that both centralized and decentralized approaches encountered similar pre-randomization challenges. Given this lack of difference in conversion rates, the total number of screened participants becomes a critical factor in determining the overall number of randomizations. In other words, models that enable broader geographic reach and greater patient access—such as DCIR—are inherently positioned to randomize more participants simply by virtue of screening more individuals.

Importantly, post-randomization discontinuation rates were also similar across all three models, with no statistically significant differences detected. The fact that DCIR sites, despite being newly established for this trial, achieved post-randomization discontinuation rates comparable to the more established traditional B&M sites is noteworthy. This parity underscores the effectiveness of DCIR sites in maintaining participant engagement and adherence, even without the longstanding operational infrastructure and historical roots of traditional sites.

### 4.3 DCIR sites replicating the success the innovation center

The Innovation Center, serving as a center of excellence and R&D hub for DCIR sites, demonstrated exceptional performance, characterized by significantly lower post-randomization discontinuation rates, as well as higher screening and randomization rates.

Building on this foundation, newly established Care Access standalone research sites (i.e., DCIR sites) have already built upon this model, demonstrating improved screening and randomization performance compared to traditional sites. These early successes validate the model’s scalability and indicate that further refinements can unlock even greater efficiencies. By continuously iterating on the lessons from the Innovation Center, the DCIR sites can push the boundaries of decentralized research, setting new benchmarks for innovation and impact at scale.

### 4.4 Operational innovations and recruitment efficiency

The randomization rate observed at Care Access sites exceeded that of non-Care Access sites, suggesting that differences in operational strategies and site models have influenced participant recruitment outcomes. This difference underscores the value of decentralized and community-integrated approaches as effective methods for addressing recruitment challenges commonly encountered in large-scale Phase 3 trials.

Several key operational innovations likely contributed to these differences in recruitment efficiency:

1. Centralized Remote Research Coordinators: This team handled remote tasks, enabling site staff to focus on participant care. This operational framework enhanced throughput, as reflected in higher screening and randomization rates across all site models. Over 60,000 participants were pre-screened across all sites, resulting in higher screening rates per site and facilitating faster participant enrollment and improved coordination across multiple locations. This centralized support for pre-screening allowed sites to redirect their capacity toward participant randomization by offloading initial participant engagement and administrative tasks. Without this centralized support, traditional brick-and-mortar sites would likely have faced even greater recruitment challenges, given the substantial screening burden.
2. Mobile Sites, Infrastructure and Traveling Staff: The use of mobile infrastructure and traveling Clinical Research Coordinators and nurses expanded the reach of decentralized sites into regions previously underserved by clinical research, ensuring broad participant accessibility without compromising trial timelines. These mobile sites initially operate within communities as flexible, mobile units. However, once they demonstrate significant promise through high utilization and strong community engagement, they can be transformed into permanent purpose-built sites, further solidifying their role in the local healthcare and research ecosystem.
3. Community Engagement: By fostering partnerships with local healthcare providers and embedding trial operations within community organizations, Care Access sites built trust and improved participant retention, particularly in populations historically underrepresented in clinical trials.

### 4.5 Costs and optimization in decentralized models

While decentralized models offer significant advantages in terms of participant outreach and recruitment flexibility, they also introduce unique cost considerations that differ from traditional B&M models.

#### 4.5.1 Participant mobility

A hallmark of the Decentralized models is the flexibility it offers participants, allowing them to participate across multiple locations. This participant mobility significantly expands the reach of recruitment efforts but also introduces logistical challenges. Mobility can introduce additional costs tied to managing transitions between locations, including added communications, and logistical support. While this flexibility enhances participant engagement, it underscores the need for robust systems to efficiently handle the added complexities without compromising the trial timeline or budget.

#### 4.5.2 Staffing, scheduling, and travel logistics

In decentralized models, travel logistics for mobile staff—such as traveling Clinical Research Coordinators and nurses—represent a major cost driver. Unlike traditional B&M models, DCIR sites often require staff to travel significant distances to conduct participant visits, administer investigational products (IP), or perform assessments. Inefficiencies in scheduling, such as sparse appointment distribution (e.g., one infusion scheduled on a Monday and another on a Friday), further exacerbate travel costs and lead to suboptimal resource utilization.

To mitigate these costs, decentralized models could benefit from regionalized staffing approaches and optimized scheduling systems. Establishing localized teams to serve nearby sites or developing regional hubs for logistical operations could significantly reduce travel demands and associated costs. Additionally, automated scheduling solutions—similar to those employed in large-scale logistics companies—could cluster participant visits geographically and temporally, ensuring more efficient coordination of staff movements and minimizing unnecessary travel. This dual approach of regional staffing and intelligent scheduling would improve cost-efficiency while maintaining the flexibility of decentralized models.

### 4.6 Comparative strengths and limitations of each site model

This trial highlighted the unique strengths and limitations of the three site models—traditional, hub-and-spoke, and DCIR—in addressing the complex demands of large-scale clinical trials. DCIR sites outperformed hub-and-spoke and traditional sites in both screening and randomization rates, driven by their community integration and purpose-built design for this trial. Unlike traditional models that require participants to travel to distant sites, DCIR sites brought research sites directly into communities that previously lacked access, enabling rapid and effective recruitment even without the benefit of a historically established network. These sites leveraged their adaptability, enabling broader geographic reach, making them particularly effective for trials requiring extensive outreach. DCIR sites maintained participant retention rates comparable to traditional sites, highlighting their ability to deliver high-quality outcomes. DCIR sites were more resource-intensive to establish and operate. To enhance cost-effectiveness, these sites could be leveraged for multiple trials and further optimized with strategies such as regionalized staffing, efficient scheduling, and the transformation of high-performing mobile units into permanent, purpose-built sites.

Hub-and-spoke models, while not as efficient as DCIR sites in screening and randomization, serve as an intermediary model that in some cases may facilitate the shift from traditional B&M setups to modern decentralized frameworks. However, this model exhibited higher variability in performance, suggesting that not all decentralized models are created equal. Unlike DCIR sites, which are designed to fully integrate research into communities, hub-and-spoke models lacked some of the key differentiators that contributed to DCIR’s success, such as deep community engagement and localized site presence.

Traditional sites, though less efficient in recruitment metrics compared to DCIR models, provide a familiar operational framework. They remain useful for trials that require frequent face-to-face interactions, regular direct patient-clinician engagements for detailed assessments, or specialized care for participants with complex therapeutic needs. However, even in these cases, incorporating elements of decentralization—such as remote follow-ups, mobile units, and home health visits—can improve accessibility without compromising clinical rigor. Additionally, traditional sites play a major role in training and mentoring new investigators, ensuring the long-term sustainability and expansion of clinical trial operations.

### 4.7 Community engagement and localized operations: the top performing community-integrated site (i.e., the innovation center) case study

The performance of Care Access’s site (i.e., the Innovation Center) underscores the significant impact that community engagement and localized operations can have on clinical trial outcomes, particularly in participant retention and randomization efficiency. The Innovation Center operated under a unique, community-integrated model. This site’s strong network of local physicians and deep-rooted ties to the community set it apart from traditional sites, positioning it as a standout example within the trial.

The Innovation Center demonstrated exceptional metrics, screening 886 participants with an average of 32.77 participants screened per month and achieving a notably low screen failure rate of 90.29%, compared to approximately 95% at other Care Access sites. Its post-randomization discontinuation rate of 17.65% was also the lowest among all sites, highlighting the effectiveness of a community-driven, participant-centered approach. These results suggest that the site’s trust-based relationships with local healthcare providers, community leaders, and participants fostered a strong sense of engagement, which helped reduce pre-randomization dropout rates and enhanced long-term retention.

However, it is important to note that the success of the Innovation Center was built over years of consistent engagement and trust within the community—a model that, while highly effective, is not readily available to newly established sites. Building such deep community ties requires sustained commitment and local integration, which may not be feasible for all trial sites, particularly in new or transient locations. However, there are valuable lessons to be learned from this approach, and newly established sites can aim to build such networks over time by leveraging this success and applying its insights. The Innovation Center’s case illustrates how combining centralized operational support with strong local relationships can amplify trial performance, particularly in complex therapeutic areas, where participant engagement and retention are challenging yet crucial.

### 4.8 Leveraging the Care Access future of medicine (FoM) health screening program for future decentralized clinical trials

The Care Access Future of Medicine (FoM) community health screening program offers another transformative addition to decentralized trials. Unlike the pre-screening conducted as part of this study, FoM operates independently as an IRB-approved research, community education, and health screening initiative. It proactively engages communities through structured outreach, health assessments, and pre-screening activities—creating new opportunities for participation by providing FoM members with medical resources, health insights, and access to clinical trials that match their health profiles, effectively bringing research directly to the communities. This approach further reduces the recruitment burden on individual sites, enhances trial readiness, and lowers access barriers—particularly among underserved populations. By maintaining a continuous pipeline of eligible FoM members, the program boosts operational efficiency and minimizes logistical challenges.

The FoM Program has the potential to provide a model to elevate the performance of existing traditional sites by adopting decentralized, community-integrated elements without requiring the creation of entirely new DCIR sites. In this context, FoM may offer a complementary solution, bringing trial components such as outreach, education, and pre-screening directly to communities. Through mobile health screenings and community engagement, FoM raises awareness and opens clear pathways to clinical trial participation, identifying and referring eligible members to nearby research sites.

The new capabilities that FoM can bring to traditional sites will enable these sites to perform more like DCIR sites. This approach not only expands geographic outreach for the traditional sites, but also fosters deeper community integration, increased engagement, and improved representation in clinical research. For trials and regions where DCIR sites are neither feasible nor necessary, FoM provides an adaptable and complementary solution that brings core trial activities—community outreach, health screenings, and education—directly to participants, broadening equitable access to research opportunities.

Collectively, these innovations highlight how existing traditional sites can leverage decentralized and community-focused methodologies, to replicate and scale operational success.

## Data Availability

The original contributions presented in the study are included in the article/supplementary material, further inquiries can be directed to the corresponding authors.
